# Toxoplasmosis is a risk factor for acquiring SARS-CoV-2 infection and a severe course of COVID-19 in the Czech and Slovak population: a preregistered exploratory internet cross-sectional study

**DOI:** 10.1186/s13071-021-05021-9

**Published:** 2021-09-28

**Authors:** Jaroslav Flegr

**Affiliations:** 1 Laboratory of Evolutionary Biology, Division of Biology, Department of Philosophy and History of Sciences, Faculty of Science, Charles University, Viničná 7, Prague 2, 128 00 Czech Republic; 2 National Institute of Mental Health, Klecany, 250 67 Czech Republic

**Keywords:** COVID-19, Risk factors, SARS-CoV-2, Symptoms, Zoonosis, Pets, Cat

## Abstract

**Background:**

Latent toxoplasmosis, i.e. a lifelong infection with the protozoan parasite *Toxoplasma gondii*, affects about a third of the human population worldwide. In the past 10 years, numerous studies have shown that infected individuals have a significantly higher incidence of mental and physical health problems and are more prone to exhibiting the adverse effects of various diseases.

**Methods:**

A cross-sectional internet study was performed on a population of 4499 (786 *Toxoplasma*-infected) participants and looked for factors which positively or negatively affect the risk of SARS-CoV-2 infection and likelihood of a severe course of COVID-19.

**Results:**

Logistic regression and partial Kendall correlation controlling for sex, age, and size of the place of residence showed that latent toxoplasmosis had the strongest effect on the risk of infection (OR = 1.50) before sport (OR = 1.30) and borreliosis (1.27). It also had the strongest effect on the risk of severe course of infection (*Tau* = 0.146), before autoimmunity, immunodeficiency, male sex, keeping a cat, being overweight, borreliosis, higher age, or chronic obstructive pulmonary disease. Toxoplasmosis augmented the adverse effects of other risk factors but was not the proximal cause of the effect of cat-keeping on higher likelihood of COVID infection and higher severity of the course of infection because the effect of cat-keeping was also observed (and in particular) in a subset of *Toxoplasma*-infected respondents (*Tau* = 0.153). Effects of keeping a cat were detected only in respondents from multi-member families, suggesting that a cat could be a vector for the transmission of SARS-CoV-2 within a family.

**Conclusions:**

Toxoplasmosis is currently not considered a risk factor for COVID-19, and *Toxoplasma*-infected individuals are neither informed about their higher risk nor prioritised in vaccination programs. Because toxoplasmosis affects a large segment of the human population, its impact on COVID-19-associated effects on public health could be considerable.

**Graphical abstract:**

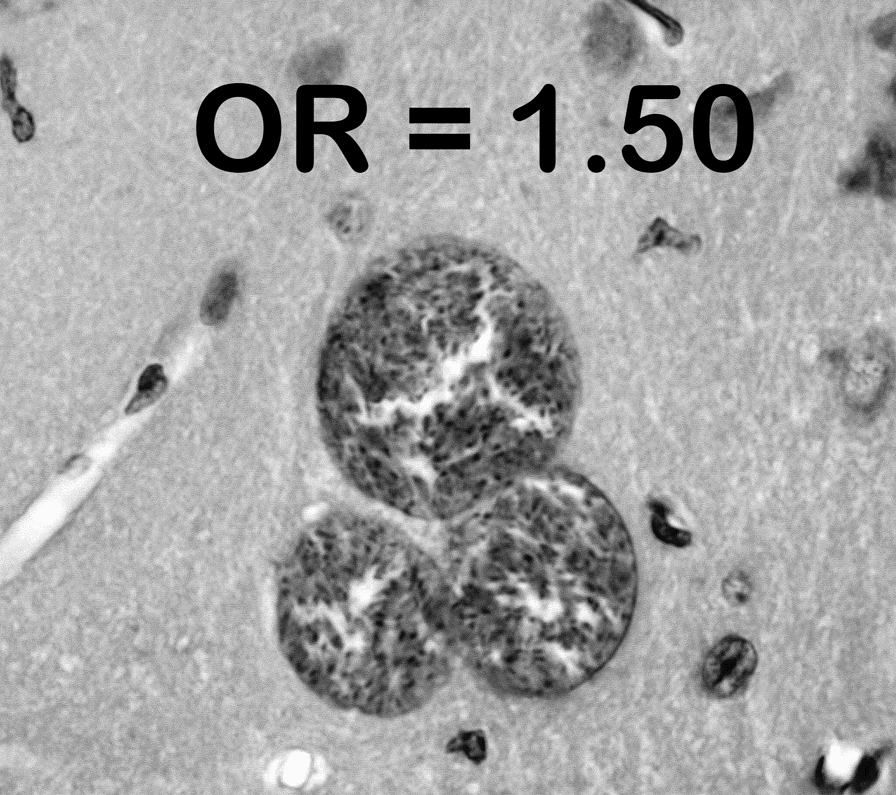

## Background

About one-third of the world’s population is infected with the protozoan parasite *Toxoplasma gondii*. The definitive host of *Toxoplasma* is cats (or any felines), but its intermediate host can be any warm-blooded animal, including humans. In immunocompetent human hosts, a short phase of acute toxoplasmosis spontaneously resolves into a latent phase. It is widely believed that during this phase, bradyzoites, the slowly reproducing stage of *Toxoplasma*, survive in tissue cysts localised mainly in immune-privileged organs and tissues for the duration of the host’s life, waiting for the host to be eaten by a cat [1]. The bradyzoites in cysts, however, can be reactivated into rapidly dividing tachyzoites in immunocompromised individuals, for example in AIDS patients or persons after transplantation or anticancer treatment [2]. This can result in the formation of brain lesions and, if untreated, lead to fatal toxoplasmic encephalitis [3].

Latent toxoplasmosis has long been considered asymptomatic in immunocompetent individuals, which is why no effort has been invested in finding a drug that would kill the bradyzoites in cysts and treat latent toxoplasmosis. This attitude is now slowly changing [4]. For example, ocular toxoplasmosis, an important cause of infectious uveitis, was believed to be associated with the relatively rare congenital toxoplasmosis [5], but current data strongly suggest that it is a relatively common effect of postnatally acquired toxoplasmosis, the most common form of toxoplasmosis. It is estimated that in Europe and the USA, about 2% of individuals with ‘latent’ toxoplasmosis have scars or active lesions on the retina [6–8]. Even more serious is the situation in South America, where there are more virulent strains of *Toxoplasma* and the highest diversity of *Toxoplasma* strains [9]. In this region, about 20% of *Toxoplasma*-seropositive individuals probably have ocular toxoplasmosis [10]. Moreover, ocular toxoplasmosis is not the only serious sequela of postnatal *Toxoplasma* infection. Latent *Toxoplasma* infection is often also associated with various inflammatory diseases [11, 12], autoimmune diseases [13], and even some types of cancer [14, 15].

Over the past 20 years, numerous studies have shown that latent toxoplasmosis has important adverse effects on the mental and physical health of infected persons [16–18]. Over 100 studies have shown that latent toxoplasmosis strongly affects (according to meta-analytic studies, nearly triples) the risk of schizophrenia. Later, a similar and possibly even stronger effect of toxoplasmosis was described in relation to other mental health disorders, including obsessive–compulsive disorder, attention-deficit/hyperactivity disorder, and autism [19, 20].

In the past decade, several studies have shown that latent toxoplasmosis affects both mental and physical health. The first evidence was merely indirect: researchers noticed that infected patients showed clear psychological symptoms of chronic stress, which explained most earlier observed differences in the personality profile and behaviour of *Toxoplasma*-infected versus *Toxoplasma*-free individuals [21]. These changes, which go in the opposite direction in men and women [22, 23] were at first interpreted as products of the parasite’s manipulative activity aimed at enhancing transmission from the intermediate host (usually a mouse) to the definitive host (a cat) by predation [24]. A large cross-sectional study performed in 333 infected participants and 1153 controls showed that *Toxoplasma*-infected individuals reported higher rates of 77 of 134 listed disorders [25]. Infected participants also scored significantly worse on 28 out of 29 health-related variables included in the study. Similar results were obtained in an ecological (correlational) study that was based on data from the World Health Organization (WHO) on the incidence of disease and disease burden in 88 WHO Member States [26]. The results showed that disability-adjusted life years (DALYs) for 23 out of 128 diseases and disease categories correlated with the prevalence of latent toxoplasmosis in individual countries even after controlling for per capita gross domestic product (GDP), latitude, and humidity. In 29 European countries, differences in the prevalence of toxoplasmosis were found to be responsible for 23% of total variability in disease burden in a model containing the three above-mentioned covariates. The strongest associations were observed with cardiovascular diseases, perinatal conditions, and congenital abnormalities (which probably reflects the effect of congenital, not latent, toxoplasmosis). Nevertheless, a strong positive association was also observed with filariasis, measles, and leukaemia. Such a broad range of observed associations suggests that the effects of latent toxoplasmosis are rather non-specific. It has also been shown that toxoplasmosis makes individuals prone to various adverse factors, including fatigue, faster aging, and smoking [27]. It is also known that *Toxoplasma* modifies the functioning of the host’s immune system, especially by increasing the concentration of certain lymphokines (most notably IL-10) [28–31] and changing the counts of various immunocytes [32].

The main aim of the present study was to explore whether latent toxoplasmosis has any effect on the risk of severe acute respiratory syndrome coronavirus 2 (SARS-CoV-2) infection and the course of coronavirus disease 2019 (COVID-19). For this purpose, the probability of being diagnosed with COVID-19, severity of the course of the disease, and the incidence of specific symptoms of the disease were compared among 4499 participants in a large internet survey.

## Methods

### General set-up

Participants were recruited by a Facebook-based snowball method [33]. Calls for participation in the study were published about 15 times on the Facebook page of Labbunnies, a 23,000-member group of Czech and Slovak nationals willing to take part in evolutionary psychology studies and help with recruiting participants for such studies, as well as on the author’s personal Facebook and Twitter accounts. The Qualtrics questionnaire that was used to gather data contained Facebook ‘share’ and ‘like’ buttons, so that participants could help recruit other participants by pressing these buttons. In total, the buttons were pressed 12,000 times between 17 October 2020 and 3 March 2021, and data from 52,000 respondents were obtained. The invitation and the informed consent form on the first page of the questionnaire contained only the most general information about the aims of the study and contents of the questionnaire. The respondents were informed that the study was examining which factors affect the risk and course of COVID-19 infection and people’s opinions about anti-epidemic measurements. Participants were also informed that their participation was voluntary, and that they could skip any questions they might find uncomfortable and terminate their cooperation at any point simply by closing the web page. Only those who consented to participate in the study by pressing the corresponding button were allowed to take the questionnaire. Respondents were not paid for their participation in the study, but after finishing the 20-minute questionnaire, they received information about the results of related studies. The study was anonymous, but participants had the option of providing their email addresses for a future longitudinal study (about 42% did) or could ask for their data to be erased after completing the questionnaire (about 2% did). Data collection was performed in accordance with the relevant guidelines and regulations, and the project, including the method for obtaining informed consent from all participants in this anonymous study, was approved by the Institutional Review Board of the Faculty of Science, Charles University (Komise pro práci s lidmi a lidským materiálem Přírodovědecké Fakulty Univerzity Karlovy)—No. 2020/25. The study was preregistered at the Open Science Framework: https://doi.org/10.17605/OSF.IO/VWXJE

### Questionnaire

The Qualtrics survey consisted of three parts related to three different projects (risk and protective factors, opinions of the Czech public on the anti-epidemic measurements, and the effect of priming by studying graphs of COVID victims on opinions regarding anti-epidemic measures). In the present study, only responses to questions related to COVID-19 risks and protective factors were inspected and analysed. Respondents were asked about their sex, age, size of their place of residence (scale 1–5, 0: under 1000 inhabitants, 1: 1000–5000 inhabitants, 2: 5000–50,000 inhabitants, 3: 50,000–100,000 inhabitants, 4: 100,000–500,000 inhabitants, 5: over 500,000 inhabitants), and how many persons lived with them in the same household. Respondents indicated whether they had already contracted COVID-19 by choosing from five answers (1: ‘No’, 2: ‘Yes, I was diagnosed with it’, 3: ‘Yes, but I was not diagnosed with it’, 4: ‘I am awaiting test results’, 5: ‘No, but I was in a quarantine’). For the purpose of the current study, answers 1 and 5 were coded as 0 (uninfected with COVID), answer 2 as 1 (COVID patients), and answers 3 and 4 as ‘na’ (data not available). The respondents were also asked whether they had ever been tested in a laboratory for toxoplasmosis and/or borreliosis and what their results were (1: ‘I do not know, I am not sure’, 2: ‘No, I was tested and the result was negative’, 3: ‘Yes, I was tested and the result was positive’). For both toxoplasmosis and borreliosis, the questionnaire was preset to indicate the first response, ‘I do not know, I am not sure’, as a default. Similarly, respondents were asked about their Rh status (positive/negative/‘I do not know, I am not sure’) and blood group (A/B/AB/O/‘I do not know, I am not sure’). They were also asked to indicate which risks and protective factors applied to them (for a list of the corresponding binary variables, see column 1 of Table 1). Those who had been diagnosed with COVID-19 were also asked to rate the severity of the course of the disease on a five-point scale (1: no symptoms, 2: like a mild flu, 3: like a severe flu, 4: I was hospitalised, 5: I was treated at an ICU [intensive care unit]). They also had to check which symptoms they experienced during the course of the COVID infection (for a list of the corresponding binary variables, see column 1 of Table 3).

**Table 1:** Incidence of risk and protective factors for COVID-19 in men and women

	Women				Men				OR	CI_95_	*P*
	No COVID		COVID		No COVID		COVID				
	*N*	%	*N*	%	*N*	%	*N*	%			
Toxoplasmosis	563	18.3	81	23.9	115	12.1	27	20.6	**1.50**	**1.19–1.89**	**0.0007**
Borreliosis	410	19.9	57	24.3	108	14.6	17	17.0	1.27	0.96–1.68	0.0881
Rh-positivity	1953	76.4	219	76.3	490	75.9	61	74.4	0.98	0.76–1.26	0.8854
A blood group	989	37.3	123	41.1	236	34.1	33	35.9	1.16	0.93–1.44	0.1795
B blood group	568	21.4	50	16.7	156	22.5	22	23.9	0.82	0.62–1.07	0.1368
AB blood group	247	9.3	34	11.4	91	13.1	13	14.1	1.19	0.86–1.65	0.2929
O blood group	850	32.0	92	30.8	210	30.3	24	26.1	0.91	0.73–1.15	0.4396
Being overweight	739	35.1	90	31.0	258	42.6	35	33.0	0.74	0.57–0.95	0.0169
Diabetes	75	3.6	7	2.4	28	4.6	8	7.6	0.96	0.52–1.76	0.8921
Cardiovascular problems	131	6.2	16	5.5	65	10.7	9	8.5	0.81	0.50–1.30	0.3820
Asthma	1846	87.7	256	88.3	544	89.9	89	84.0	1.13	0.80–1.59	0.4908
Chronic obstructive pulmonary disease	49	2.3	5	1.7	16	2.6	2	1.9	0.67	0.27–1.63	0.3737
Immunosuppression	289	13.7	40	13.8	52	8.6	9	8.5	1.00	0.70–1.42	0.9929
Allergy	548	26.0	77	26.6	150	24.8	28	26.4	1.06	0.81–1.37	0.6798
Autoimmunity	168	14.4	25	11.6	26	8.7	6	9.1	0.75	0.45–1.24	0.2545
Living alone	285	9.3	28	8.3	160	16.9	23	17.6	0.96	0.70–1.32	0.8027
Tobacco smoking	415	19.7	54	18.6	148	24.5	23	21.7	0.89	0.66–1.19	0.4341
Marijuana consumption	56	2.7	6	2.1	40	6.6	14	13.2	1.49	0.88–2.53	0.1367
Daily alcohol consumption	126	6.0	16	5.5	97	16.0	14	13.2	0.86	0.55–1.33	0.4887
Frequent singing	269	12.8	39	13.5	50	8.3	14	13.2	1.21	0.87–1.70	0.2612
Sport	620	29.5	92	31.7	274	45.3	60	56.6	1.30	1.03–1.65	0.0300
Cold water swimming	205	9.7	37	12.8	102	16.9	19	17.9	1.32	0.95–1.84	0.0989
Frequent sauna use	94	8.0	20	9.3	37	12.4	8	12.1	1.16	0.70–1.93	0.5558
Vitamins and supplements	753	35.8	133	45.9	289	47.8	60	56.6	**0.62**	**0.49–0.78**	**0.0001**
Wearing glasses	1101	60.4	169	61.9	371	67.8	67	66.3	0.96	0.75–1.24	0.7723
Dog-keeping	365	44.2	78	49.4	93	51.1	21	45.7	0.82	0.56–1.18	0.282
Cat-keeping	381	46.1	82	51.9	82	45.1	17	37.0	1.16	0.80–1.68	0.4303

The last four columns show the results of logistic regression with COVID-19 infection as a dependent variable; age, sex, and size of the place of residence as covariates; and one of risk/protective factors listed in the first column as the independent variable. Results shown in bold were significant after correction for multiple tests

*CI*_*95*_ means 95% confidence interval and *OR* odds ratio

### Statistical analyses

Before embarking on any analyses, all participants who completed the questionnaire for the second or third time (they were asked about this at the end of the questionnaire for purposes of the planned longitudinal study), those who answered all or nearly all questions with the same code, and those who finished the questionnaire in less than 6 minutes were filtered out. After this filtering, the set contained 29,345 records. Only participants who tested negative or positive for toxoplasmosis (4499 participants) were included in the present study.

Statistical analysis was performed with the Statistica v.10.0. (descriptive statistics, *t*-tests) and R v.3.3.1 [34] (all other tests) packages. To compute the partial Kendall correlation, the ppcor package [35] and the Explorer 1.0 package were used [36]. Correction for multiple tests was done using the Benjamini–Hochberg procedure with the false discovery rate preset to 0.10 [37].

## Results

The final set contained 1085 men, 142 (13.1%) of whom were infected with *Toxoplasma* and 131 (12.1%) diagnosed with COVID, and 3414 women, 644 (18.9%) of whom were infected with *Toxoplasma* and 339 (9.9%) diagnosed with COVID. The incidence of other potential risk factors is shown in Table 1.

A significant difference in age between men (38.9, standard deviation [SD] 10.9) and women (40.0, SD 10.5) (*t*_(4497)_ = −2.86, *P* = 0.0043) and between *Toxoplasma*-free (39.3, SD 10.5) and *Toxoplasma*-infected (42.7, SD 10.1) women (*t*_(3412)_ = −7.41, *P* < 0.0001), but not between *Toxoplasma*-free (38.7, SD 10.7) and *Toxoplasma*-infected (40.0, SD 11.8) men (*t*_(1083_ = −1.25, *P* = 0.211), were found in the population under study. No association between age and COVID was detected in either men or women (*P* > 0.38).

The risk of acquiring toxoplasmosis depends on certain confounding factors, such as sex, age, and size of the place of residence. Therefore, the association between potential risk factors and protective factors was analysed using a logistic regression with binary variable COVID as a dependent variable; sex, age, and size of the place of residence as covariates; and one of the analysed factors as the independent variable. The results are shown in the last three columns of Table 1. Toxoplasmosis, with an odds ratio of 1.50 (95% confidence interval [CI_95_] = 1.19–1.89, *P* = 0.0007, odds ratio 1.40 (CI_95_: 1.05–1.80) for women and 1.91 (CI_95_: 1.20–3.05) for men, turned out to be the most serious risk factor for being diagnosed with COVID.

It is known that Rh-negative individuals are more prone to experiencing the negative effects of toxoplasmosis than those who are Rh-positive [38–40]. Therefore, in addition, a more complex model containing not only sex, age, size of the place of residence, and toxoplasmosis, but also the Rh factor and Rh–toxoplasmosis interaction, was analysed. Logistic regression showed a significant effect of toxoplasmosis (odds ratio [OR] = 1.45, CI_95_: 1.06–2.10, *P* = 0.046), but not Rh (*P* = 0.86) or Rh–toxoplasmosis interaction (*P* = 0.84).

**Fig. 1:**
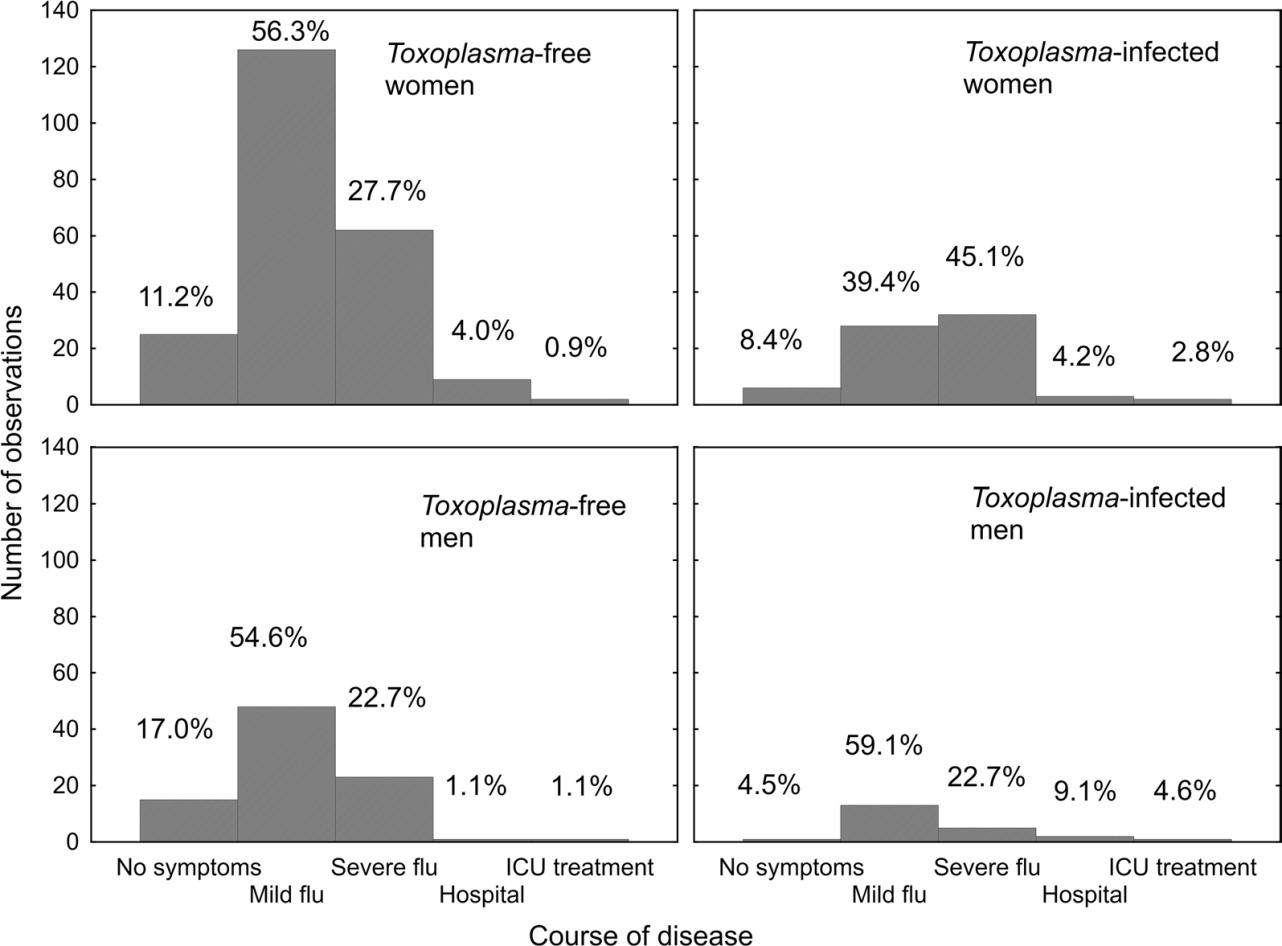
The course of COVID-19 in *Toxoplasma*-free and *Toxoplasma*-infected men and women

Respondents who had been diagnosed with COVID-19 were asked to rate the course of their disease and indicate which symptoms they experienced during their illness. Figure 1 shows that *Toxoplasma*-infected participants of both sexes had a more serious course of COVID. Partial Kendall correlation (Table 2) showed that toxoplasmosis represents a more pronounced risk factor for a severe course of COVID-19 than does compromised autoimmunity, immunodeficiency, male sex, cat-keeping, being overweight, borreliosis, higher age, or chronic obstructive pulmonary disease.

**Table 2:** Correlation between various risk/protective factors and severity of COVID-19

	All		Women		Men		Toxo-free		Toxo-infected	
	*Tau*	*P*	*Tau*	*P*	*Tau*	*P*	*Tau*	*P*	*Tau*	*P*
Sex	**0.116**	**0.0002**	na	na	na	na	**0.112**	**0.0017**	0.095	0.1713
Age	**0.093**	**0.0032**	**0.124**	**0.0010**	0.025	0.6763	0.066	0.0654	0.137	0.0494
Size of place of residence	0.009	0.7815	−0.002	0.9575	0.039	0.5093	0.000	0.9895	0.065	0.3477
Toxoplasmosis	**0.146**	**0.0000**	**0.150**	**0.0001**	0.123	0.0376	na	na	na	na
Borreliosis	**0.094**	**0.0123**	**0.120**	**0.0088**	0.030	0.6529	0.010	0.8146	0.212	0.0301
Rh-positivity	0.036	0.3097	0.035	0.3929	0.064	0.3950	0.022	0.5941	0.098	0.2112
A blood group	0.019	0.5892	0.000	0.9945	0.079	0.2715	0.032	0.4210	−0.052	0.4932
B blood group	−0.008	0.8101	−0.010	0.7940	0.009	0.8991	0.012	0.7680	−0.069	0.3606
AB blood group	0.021	0.5371	**0.093**	**0.0210**	−0.160	0.0254	−0.002	0.9586	0.080	0.2909
O blood group	−0.028	0.4278	−0.050	0.2142	0.031	0.6694	−0.043	0.2834	0.048	0.5260
Being overweight	**0.104**	**0.0012**	**0.107**	**0.0052**	0.116	0.0558	0.089	0.0147	0.140	0.0505
Diabetes	0.004	0.8922	−0.021	0.5777	0.038	0.5301	0.019	0.5929	−0.022	0.7602
Cardiovascular problems	0.063	0.0514	0.063	0.0985	0.075	0.2194	0.049	0.1762	0.170	0.0174
Asthma	0.052	0.1055	0.036	0.3496	0.094	0.1233	0.039	0.2815	0.133	0.0630
Chronic obstructive pulmonary disease	**0.072**	**0.0252**	0.073	0.0583	0.080	0.1853	0.013	0.7220	**0.219**	**0.0022**
Immunodeficiency	**0.135**	**0.0000**	**0.141**	**0.0002**	0.116	0.0558	**0.160**	**0.0000**	0.067	0.3472
Allergy	−0.015	0.6453	−0.011	0.7829	−0.025	0.6809	−0.028	0.4376	0.025	0.7235
Autoimmunity	**0.137**	**0.0004**	**0.146**	**0.0011**	0.112	0.1590	0.074	0.0874	*0.254*	*0.0048*
Living alone	0.034	0.2794	**0.092**	**0.0151**	−0.061	0.2999	0.030	0.3960	0.073	0.2990
Tobacco smoking	0.061	0.0576	0.045	0.2454	0.090	0.1364	0.082	0.0239	0.006	0.9351
Marijuana consumption	−0.001	0.9777	**−0.082**	**0.0330**	0.081	0.1800	0.057	0.1207	**−0.195**	**0.0065**
Daily alcohol consumption	−0.039	0.2311	**−0.094**	**0.0138**	0.067	0.2677	−0.055	0.1279	0.035	0.6289
Frequent singing	−0.032	0.3162	−0.060	0.1153	0.056	0.3553	−0.053	0.1459	−0.001	0.9861
Sport	**−0.092**	**0.0044**	**−0.112**	**0.0036**	−0.056	0.3543	**−0.116**	**0.0015**	−0.028	0.6944
Cold water swimming	−0.047	0.1447	−0.041	0.2851	−0.055	0.3614	−0.062	0.0883	−0.041	0.5691
Frequent use of sauna	−0.036	0.3550	−0.040	0.3731	−0.037	0.6432	−0.009	0.8280	−0.177	0.0491
Vitamins and supplements	−0.047	0.1425	−0.029	0.4437	−0.087	0.1499	−0.068	0.0628	0.034	0.6329
Wearing glasses	−0.033	0.3244	0.005	0.9037	−0.131	0.0346	−0.003	0.9346	−0.069	0.3559
Current dog-keeping	0.004	0.9321	0.015	0.7737	−0.042	0.6694	0.049	0.3497	−0.120	0.2637
Current cat-keeping	**0.107**	**0.0203**	**0.123**	**0.0194**	0.058	0.5549	0.084	0.1052	0.153	0.1544

The table shows effect size and significance, i.e. partial Kendall *Tau* and *P*-values. The effect of sex was controlled for age and size of place of residence, the effect of age for sex and size of place of residence, and size of place of residence for sex and age; all other effects were controlled for all three covariates. Positive *Tau* indicates a more severe course of COVID in participants reporting the factor listed in the first column. The results in bold were significant after correction for multiple tests. In the smaller group of men, all factors lost their significance after correction for multiple tests, but observed *Tau* values showed that the effects in men were generally stronger than those in women

One could speculate that keeping a cat could just be a proxy for being *Toxoplasma*-infected, and indeed, the prevalence of toxoplasmosis in participants who did keep a cat (24.6%) was markedly higher than in those who did not (15.5%) (χ^2^ = 15.5, *P* < 0.0001). To test this hypothesis, the partial Kendall correlation test was performed separately for *Toxoplasma*-free and *Toxoplasma*-infected participants (Table 2, the last four columns). The existence of cat-keeping effects (i.e. increased likelihood of contracting COVID-19 and of having a severe course of the disease in the cat-keepers) in the *Toxoplasma*-infected subpopulation suggests that keeping a cat is a real risk factor for COVID-19, not just a side effect of a higher probability of having toxoplasmosis. In fact, the strength of the cat-keeping effect was twice as high in the *Toxoplasma*-infected (*Tau* = 0.153) as the *Toxoplasma*-free participants (*Tau* = 0.084). One could speculate that a cat might be able to transmit COVID among family members. Members of one family could moreover be infected repeatedly, and the virus could adapt to the similar genotype of (genetically related) members of the same family; both of the above scenarios could then result in a more severe course of the disease. Indeed, separate analyses for 194 respondents who lived in multi-member families showed that keeping a cat increased the risk of a severe course of COVID-19 (*Tau* = 0.116, *P* = 0.017). The same analysis for 22 respondents who lived alone showed no such increase; in fact, it showed a non-significant effect in the opposite direction (*Tau* = −0.028, *P* = 0.867).

Analogical analyses of the risk of acquiring SARS-CoV-2 infection showed no significant effect of cat-keeping either in people who lived in multi-member families or in those who lived alone. However, even here a positive non-significant effect of cat-keeping was found in 3983 respondents living in multi-member families (OR = 1.210, CI_95_: 0.82–1.78, *P* = 0.332), and a negative non-significant effect was found in 496 respondents living alone (OR = 0.692, CI_95_: 0.20–2.46, *P* = 0.568).

The results also showed that many factors taken into consideration in this study had a much stronger effect on the risk of severe course of COVID in *Toxoplasma*-infected participants than in those who were *Toxoplasma*-free: borreliosis (*Tau* = 0.212 vs 0.010), being overweight (*Tau* = 0.140 vs 0.089), cardiovascular diseases (*Tau* = 0.170 vs 0.049), asthma (*Tau* = 0.133 vs 0.039), chronic obstructive pulmonary disease (*Tau* = 0.219 vs 0.013), allergy (*Tau* = 0.089 vs 0.012), compromised autoimmunity (*Tau* = 0.254 vs 0.074), and cat-keeping (*Tau* = 0.153 vs 0.0840). Conspicuous exceptions were male sex (*Tau* = 0.095 vs 0.112) and immunodeficiency (*Tau* = 0.067 vs 0.160). The protective effect of sport was also stronger in the *Toxoplasma*-free than in the *Toxoplasma*-infected subset (*Tau* = −1.16 vs 0.028).

Logistic regression controlled for age, sex, and size of the place of residence showed that *Toxoplasma*-infected participants reported having more symptoms during COVID, but only the presence of chest pain or pressure (OR = 1.96) and higher sexual desire (OR = 2.68) were statistically significant after correction for multiple tests (Table 3). On the other hand, even the effects that did not reach the formal level of statistical significance tended to show a higher frequency of symptoms in *Toxoplasma*-infected than in those who were *Toxoplasma*-free, and were nearly always stronger in men than in women.

**Table 3:** Symptoms of COVID-19 in *Toxoplasma*-infected and *Toxoplasma*-free participants

	All		Women		Men		OR				
	Toxo−	Toxo+	Toxo−	Toxo+	Toxo−	Toxo+		CI_95_	*P*	OR_W_	OR_M_
Fever > 38 °C (100.4 °F)	0.38	0.39	0.34	0.37	0.47	0.45	1.07	0.67–1.7	0.789	1.09	0.99
Fatigue	0.74	0.81	0.79	0.90	0.57	0.57	1.19	0.45–3.14	0.729	1.30	1.22
Dry cough	0.42	0.53	0.44	0.50	0.38	0.64	1.40	0.89–2.2	0.141	1.16	2.97
Shortness of breath	0.32	0.39	0.33	0.41	0.31	0.32	1.19	0.74–1.91	0.466	1.30	1.04
Sore throat	0.36	0.36	0.37	0.40	0.34	0.23	1.00	0.63–1.61	0.985	1.08	1.36
Headache	0.70	0.78	0.74	0.84	0.60	0.59	1.37	0.82–2.29	0.225	1.71	0.90
Chest pain or pressure	0.32	0.51	0.35	0.53	0.24	0.45	**1.96**	**1.24–3.13**	**0.004**	1.79	2.73
Other pains	0.31	0.38	0.33	0.41	0.26	0.27	1.20	0.74–1.93	0.453	1.16	1.18
Diarrhoea	0.28	0.36	0.29	0.36	0.25	0.36	1.40	0.86–2.26	0.171	1.17	2.26
Conjunctivitis	0.08	0.12	0.09	0.14	0.05	0.05	1.28	0.6–2.72	0.522	1.22	1.84
Loss of smell	0.71	0.72	0.73	0.74	0.66	0.64	0.99	0.61–1.59	0.954	0.99	0.94
Loss of taste	0.58	0.55	0.62	0.59	0.49	0.45	0.88	0.56–1.39	0.593	0.89	0.87
Middle ear pain	0.12	0.22	0.14	0.25	0.06	0.14	1.95	0.68–5.56	0.209	1.67	5.11
Problems speaking and walking	0.03	0.05	0.03	0.06	0.04	0.05	1.72	0.6–4.98	0.313	1.32	2.18
Skin rash	0.08	0.10	0.10	0.09	0.02	0.14	1.31	0.6–2.87	0.492	0.77	6.00
Changes in pigmentation	0.03	0.05	0.04	0.06	0.01	0.05	1.81	0.64–5.09	0.262	1.29	4.05
Higher sexual desire	0.03	0.08	0.03	0.06	0.04	0.14	**3.40**	**1.42–8.17**	**0.006**	2.53	4.50
Other symptoms	0.27	0.25	0.29	0.27	0.22	0.18	0.93	0.56–1.55	0.778	0.90	0.99

Columns 2–7 show the reported incidence of particular symptoms (in percentages); the last five columns show the results of logistic regression with a particular symptom as the dependent variable; age, sex, and size of place of residence as covariates; and toxoplasmosis as the independent variable. The last two columns show the odds ratio computed separately for women (OR_W_) and men (OR_M_). The results in bold were significant after correction for multiple tests

## Discussion

*Toxoplasma*-infected participants had a higher probability of being diagnosed with COVID-19 and of having a more severe course of the disease; they were more likely to end up hospitalised and more frequently needed to be treated at intensive care units. They more frequently described the specific COVID symptoms, especially chest pain and pressure. Surprisingly, *Toxoplasma*-infected participants also more frequently reported higher sexual desire (*P* = 0.006) during COVID-19; this indicator of the faster life strategy of individuals with impaired health [41] might deserve special attention in future studies.

The effects of toxoplasmosis on the risk of SARS-CoV-2 infection and risk of severe course of COVID-19 were the strongest effects detected in the present study. It must be remembered, however, that individuals with various known risk factors, such as being overweight, are probably more careful and actively try to avoid possible sources of the infection. It is important to note that most factors known to be linked to a severe course of COVID decreased (albeit usually non-significantly) the risk of becoming infected. *Toxoplasma* infection is not, however, considered a factor that could increase the risk of severe COVID, and *Toxoplasma*-infected persons therefore would likely not modify their behaviour to minimise the risk of becoming infected with COVID. Consumption of vitamins and food supplements seems to have had the strongest protective effect on the risk of becoming infected with COVID, but people who take vitamins probably also apply other measures to avoid the infection, which is why it cannot be determined whether vitamins and supplements alone have such a strong protective effect. Sport had the strongest protective effect against a severe course of COVID-19, but it is also a strong risk factor for acquiring the infection, probably because sport activities increase the number of contacts with potential sources of SARS-CoV-2 infection.

Our data showed that latent toxoplasmosis was the strongest risk factor for a severe course of COVID-19. It was stronger than the effect of being overweight, cardiovascular disease, or diabetes. Nevertheless, the relative strength of the particular effects should be interpreted with caution. It is possible that individuals with the most severe course of COVID (and logically also those who died) did not participate in our questionnaire study. This preselection could probably explain the relatively weak effect of chronic obstructive pulmonary disease.

There has been much speculation and some indications to the effect that pets could be a vector of COVID-19 [42–44]. Empirical data show that cats and dogs can and do acquire symptomatic or asymptomatic SARS-CoV-2 infection [45, 46]. For example, a study performed in Minnesota, USA [47] showed seroprevalence of 8% and 1% in 239 pet cats and 510 pet dogs, respectively, while a German study showed that seroprevalence in cats during the first wave of COVID was 0.7%, and increased to 1.4% after the second wave [48]. The prevalence in cats was higher in families of COVID-19 patients [49], and there is even direct evidence of human-to-cat transmission within a family [50, 51]. On the other hand, it seems that transmission of the virus between pets occurs rarely, if at all. Similarly, there is no direct evidence for transmission of SARS-CoV-2 from cats or dogs to humans [52]. It has been suggested that pets might transmit the virus at least within a family, either from infected animals themselves or by touching, e.g., stroking, healthy animals with virus-contaminated fur or paws [53]. Our data seem to support this possibility. Only a non-significant positive effect of cat-keeping was present in respondents from multi-member families, and a negative, though again non-significant, effect of cat-keeping was observed in respondents who lived alone. However, keeping a cat was found to increases the risk of a severe course of COVID: it was the fifth strongest factor after toxoplasmosis, diagnosed immunodeficiency, autoimmunity, and male sex. The existence of this effect in a subpopulation of *Toxoplasma*-infected individuals invalidates the hypothesis that cat-keeping is only a proxy of *Toxoplasma* infection (and possibly a better proxy than the presence of anti-immunoglobulin G [IgG] antibodies).

Analyses performed separately on the *Toxoplasma*-free and *Toxoplasma*-infected participants also showed that most factors had a much stronger effect (or existed only) in the *Toxoplasma*-infected subpopulation. For example, the effect of borreliosis was 21.5 times, chronic obstructive pulmonary disease 16.8 times, cardiovascular diseases 3.5 times, compromised autoimmunity 3.4 times, and age 2.1 times as strong in the *Toxoplasma*-infected as in *Toxoplasma*-free participants. This enhancing effect of toxoplasmosis also applies to some protective factors: visits to saunas and marijuana consumption protected *Toxoplasma*-infected but not *Toxoplasma*-free participants from infection. In contrast, the protective effect of sport was about four times as strong in *Toxoplasma*-free as in *Toxoplasma*-infected participants. The most conspicuous amplifying effect of toxoplasmosis was on the borreliosis–COVID interaction. The effect of being diagnosed with borreliosis was the third strongest effect observed in *Toxoplasma*-infected participants (*Tau* = 0.212), while in *Toxoplasma*-free individuals, no such effect was observed (*Tau* = 0.010). The same phenomenon has previously been observed for the interaction between toxoplasmosis, borreliosis, and depression [54]. A cross-sectional study showed that borreliosis had a relatively strong effect on reported depression, but was observed only in *Toxoplasma*-infected participants. Generally, latent toxoplasmosis seems to make human hosts more prone to a wide spectrum of adverse effects, including genetic factors and pathogens.

One can only speculate about the mechanism by which toxoplasmosis influences the risk of SARS-CoV-2 infection and the severity of the course of COVID, but it is likely that immunomodulation and immunosuppression associated with *Toxoplasma* infection play an important role [28–31]. Toxoplasmosis has a strong effect on the concentration of various cytokines, especially interleukin (IL)-10, IL-5, IL-6, and transforming growth factor beta (TGF-β). Most studies investigating this issue were performed in laboratory animals and might reflect changes associated with acute or post-acute toxoplasmosis rather than its latent stage, but some studies suggest the existence of specific changes in immunity in humans. It has been shown that many parameters among people with latent toxoplasmosis differ from those with *Toxoplasma* infection [32]; for example, seropositive women have increased levels of IL-5 and IL-6, and especially (fivefold) IL-12, in comparison to seronegative controls [55]. In immunology outpatients, *Toxoplasma*-infected women were found to have increased, and men decreased, counts of leukocytes, natural killer (NK) cells, and monocytes in comparison to corresponding *Toxoplasma*-free controls [32]. A cross-sectional study performed in a Czech population showed that *Toxoplasma*-infected participants more frequently reported immune disorders, especially immunodeficiency [25].

The main limitation of the present study is that the participants were self-selected and probably do not represent a typical Czech population. Social networks, namely Facebook and Twitter, were used to promote the study, which is why it is likely that only individuals who had internet access and used these social networks took part in the study. Moreover, many were recruited via the Facebook site of the main investigator, which narrows the profile of likely participants even further to people interested in biological sciences, evolutionary psychology, and quite possibly also in keeping cats as pets. On the other hand, they also invited their Facebook friends to participate in the study by pressing the share or like button at the end of the questionnaire. Our previous results showed that the composition of participants of similar internet studies was highly similar, if not identical, to a representative internet population with respect to the prevalence of 24 neuropsychiatric disorders [56] or religiosity [57]. It is important to emphasise that the informed consent form, as well as the text used for recruitment of participants, mentioned only ‘factors that might influence the risk of infection and the course of COVID disease’; that is, it made no reference to toxoplasmosis.

Toxoplasmosis status was self-reported by respondents, which may be viewed as a drawback. On the other hand, it was previously demonstrated that information on toxoplasmosis status provided by 3827 participants in another internet study corresponded nearly perfectly (99.5%) to information in our files on individuals tested for toxoplasmosis in our lab [58]. Still, about 60% of male and 70% of female respondents recruited via the Facebook-based snowball method were tested for toxoplasmosis elsewhere, mostly in relation to their health problems (49.4% of men) or pregnancy (37.6% of women) [59]. It is possible that some participants misreported their *Toxoplasma* status. Similarly, some respondents who were *Toxoplasma*-negative during their serological test may have acquired the infection in the meantime. It must be emphasised, however, that the presence of misdiagnosed individuals in the population can result in a type 2—not a type 1—error; that is, it can increase the risk of failure to detect existing effects but not the risk of detecting non-existent effects.

The strength of the study was the large number of participants and the fact that it was preregistered before the start of data collection. Technically, therefore, it was designed as a cross-sectional study. On the other hand, COVID-19 is a new disease, and infected Czech and Slovak participants probably acquired the infection in autumn 2020, i.e., after being diagnosed with toxoplasmosis. The study thus in fact had the nature of a prospective case–control study and can say at least something about the causal relation between toxoplasmosis and COVID.

## Conclusions

This was an exploratory, albeit preregistered and large, study performed on a rather specific subpopulation. All results must therefore be regarded as merely preliminary and they must be confirmed by future independent confirmatory studies. The main result of the present study that deserves special attention is the identification of a new risk factor for SARS-CoV-2 infection and severe course of COVID-19. That factor, latent toxoplasmosis, seems to have a stronger effect than most others known to affect the risk of COVID-19. Moreover, it seems to enhance the negative effects of certain other adverse factors, some of which had not been suspected of having any impact on the course of COVID, such as borreliosis and keeping a cat. The effect of toxoplasmosis is probably rather non-specific, akin to what has been observed for other diseases and disorders. It is probably related to the observed changes in the immune system of *Toxoplasma* hosts. It can only be speculated whether the effects, such as the highly increased level of immunosuppressive cytokine IL-10, are just a side effect of a latent lifelong infection or part of *Toxoplasma*’s biological adaptations aimed at surviving the attacks of the host’s immune system. Latent toxoplasmosis affects about one-third of the population in both the developed and developing world. The adverse effects of this zoonosis on public health are therefore probably not negligible.

## Data Availability

The dataset supporting the conclusions of this article is available in the Figshare repository [https://doi.org/10.6084/m9.figshare.14559993.v1] [60].
